# A Study of Practical Parameters and Their Relative Importance as Perceived by Various Stakeholders in Clinical Trials

**DOI:** 10.4103/0975-1483.76421

**Published:** 2011

**Authors:** R Pant, Y Joshi

**Affiliations:** *Himalayan Institute of Pharmacy & Research, Atak Farm, Rajawala, via Prem Nagar, Dehradun (Uttarakhand), India*

**Keywords:** Contract research organizations, investigators, sponsors, stakeholders

## Abstract

A contract research organization (CRO) is a company which conducts a Good Clinical Practice (GCP) in clinical trial. There are literally hundreds of CROs worldwide employing a workforce of nearly 100,000 professionals. The project proposes the study of practical parameters and their relative importance as perceived by the various stakeholders in clinical trials. The survey was conducted in Bangalore and New Delhi. Primary market data was obtained by primary market research which included 80 clinical trial stakeholders by having a preliminary communication with them, followed by administering a questionnaire along with prior permission. There were 15 Sponsors/ CROs, 27 Investigators /Monitors and 38 Ethics committee members involved in the study. It was shown from the study that a clinical investigator involved in a clinical trial is responsible for ensuring that an investigation is conducted according to the signed investigator statement, the investigational plan, and applicable regulations; for protecting the rights, safety, and welfare of the subjects under the investigator’s care; and for the control of drugs under investigation. It was also shown from the study that the sponsors of a clinical trial carry the ultimate responsibility for the initiation, management and financing of the clinical trial. The study has identified a specific training need at the level of the individual stakeholder to perform a particular job function and to identify the actual practical parameters in the Indian context important for the conduction of clinical trials (GCP) with respect to the different stakeholders, to determine the relative importance of these parameters as perceived by various stakeholders involved in clinical trials, and to identify the relative contributions of different stakeholders to the success/ satisfactory conduct of a clinical trial.

## INTRODUCTION

A Contract Research Organization (CRO) is an organization that offers clients a wide range of pharmaceutical research services to aid in the drug and medical device research and development process.[1,2] In the Code of Federal Regulations (CFR), the USFDA regulations state that a CRO is “a person (i.e. a legal person, which may be a corporation) that assumes, as an independent contractor with the sponsor, one or more of the obligations of a sponsor, e.g., design of a protocol, selection or monitoring of investigations, evaluation of reports, and preparation of materials to be submitted to the Food and Drug Administration” [21 CFR 312.3(b)].[2] Although a set of guidelines that governs the conduct of clinical trials in any country is available nowhere is any data available that talks about the motivators/demotivators to various stakeholders (investigators/ sponsors/ ethics committee members etc.) for conducting a clinical trial.[3] This aspect is especially important for India as more and more number of studies are being undertaken in India and the clinical research profession is expected to boom. The foreign pharmaceutical companies and CROs are expanding their base to India and conducting a lot of clinical studies, however, none of them try to address the actual practical parameters that are of relative importance to Indian investigators, such as relevance of the study to the Indian population, publication policy, trial management capabilities, benefit to study participants, communication skills, grants and payments, etc.[4] Thus the study is not only an attempt to identify the practical parameters of importance for the conduct of GCP clinical trials but also to identify their relative importance in the mind of various stakeholders (investigators/ sponsors/ ethics committee members etc.). As a result it will further support the clinical research industry in India by providing the level of importance for the individual parameter. The main objectives of this study were to identify the actual practical parameters in the Indian context important for the conduction of clinical trials (GCP) with respect to the different stakeholders, to determine the relative importance of these parameters as perceived by various stakeholders involved in clinical trials and also to identify the relative contributions of different stakeholders to the success/ satisfactory conduct of a clinical trial.

## METHODOLOGY

### Source of data

The sources for market research involve both primary and secondary sources. Primary sources include Ethics Committee Members (ECMs), Investigators and Sponsors while secondary sources involve the use of Internet, Textbooks and Journals (Such as Pharma pulse, Pharma biz etc).

### Methods of data collection

Preliminary communication with various clinical research professionals/stakeholders; followed by administering a questionnaire.

### Place and time of survey

The survey was conducted in Bangalore and New Delhi from January to June 2007.

### Sample size

1. Total sample size - 80
2. Sponsors / CROs - 15
3. Investigators / Monitor - 27
4. Ethics committee members (ECMs) - 38

### *Sampling technique* - Convenience sampling

*Data analysis method*- The data obtained was analyzed using Microsoft Excel and various observations were analyzed to arrive at the conclusion.

## RESULTS

**Figure 1:**
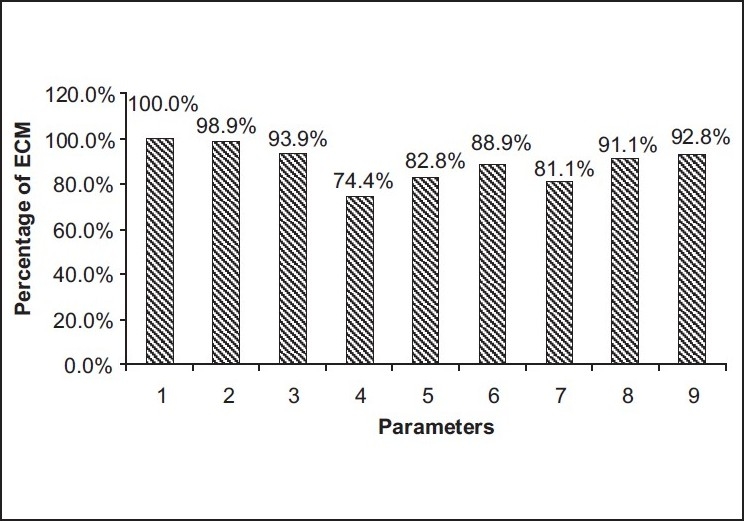
Parameters to conduct GCP clinical trials

I. Parameters to conduct GCP clinical trial according to the ECMs’ opinion [Figure 1]:

1. 100.0% mentioned scientific rationale of the study
2. 98.9% opined protection of rights and safety of the trial participants
3. 93.9% stated about the clearance of the study project from Drugs Controller General of India (DCGI)
4. 92.8% referred to investigator’s qualification and clinical research experience
5. 91.1% mentioned quality of protocol, case report form (CRF) and informed consent document (ICD) design
6. 88.9% declared compensation clause for trial-related injuries
7. 82.8% stated about the clearance of project from foreign regulatory authorities
8. 81.1% mentioned investigator’s grants and payment
9. 74.4% referred to the involvement of investigator site in the multi-countries project

II. Parameters for the role of the ethics committee in clinical triasl according to the ECMs’ opinion:

1. 100.0% mentioned safeguarding the right, safety, and wellbeing of all trial subjects
2. 92.2% stated that a competent review of all ethical aspects of the project proposals received must be ensured
3. 91.8% declared that the project be executed free from any bias and influence that could affect its objectivity
4. 90.2% opined that universal ethical values and international scientific standards should be expressed in terms of local community values and customs

III. Principles to conduct GCP clinical trial according to the ECMs’ opinion:

1. 100.0% opined principles of compliance
2. 95.6% mentioned principles of privacy and confidentiality
3. 94.5% stated principles of voluntariness, informed consent and community agreement
4. 92.9% declared principles of essentiality, and principles of totality of responsibility

**Figure 2:**
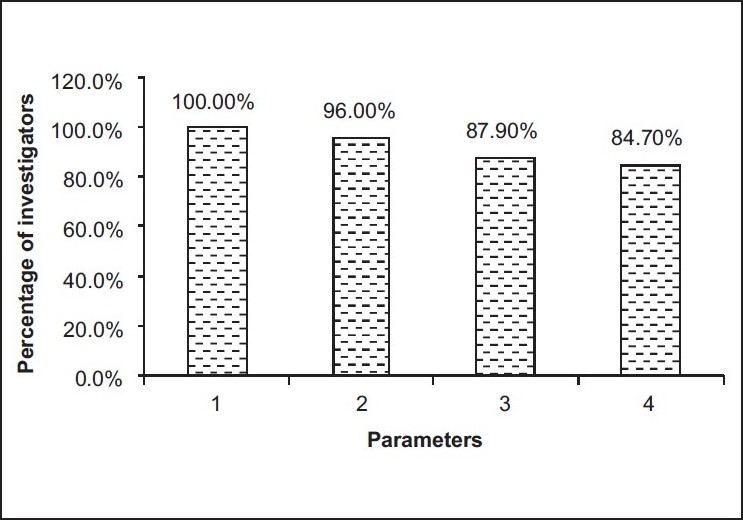
Parameters of study design to conduct GCP clinical trials

IV. Parameters of study design to conduct GCP clinical trial according to the Investigators’ opinion [Figure 2]

1. 100.0% mentioned scientific rationale of the study
2. 96.0% stated application of the study in the Indian context
3. 87.9% opined benefit to study participants
4. 84.7% declared publication policy

V. Parameters of project management (Sponsor/CRO) to conduct GCP clinical trial according to the Investigators’ opinion:

1. 100.0% opined scientific quality of protocol, CRF and ICD design
2. 96.6% mentioned trial logistics (centralized lab, imaging and data management facility)
3. 94.1% stated study grant
4. 93.3% declared inventory planning and management for study supplies

**Figure 3:**
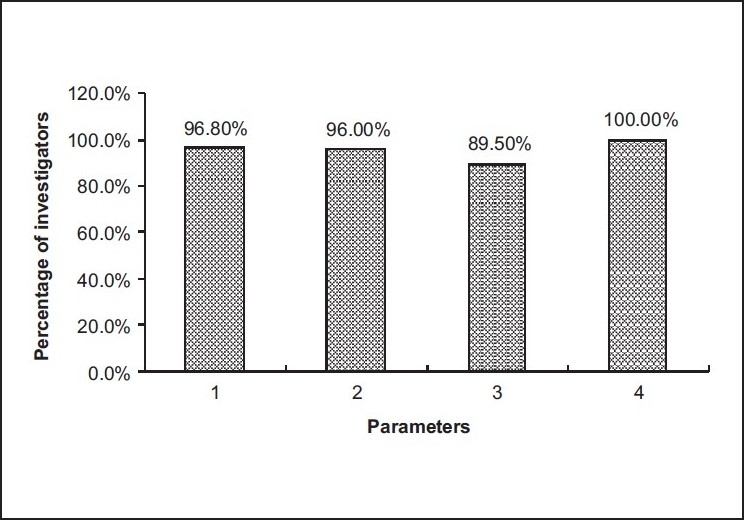
Parameters of personnel quality to conduct GCP clinical trial

VI. Parameters of personnel quality to conduct GCP clinical trial according to the Investigators’ opinion [Figure 3]:

1. 100.0% mentioned trial management capabilities
2. 96.8% declared qualification and experience
3. 96.0% opined communication skills
4. 89.5% stated responsiveness

**Figure 4:**
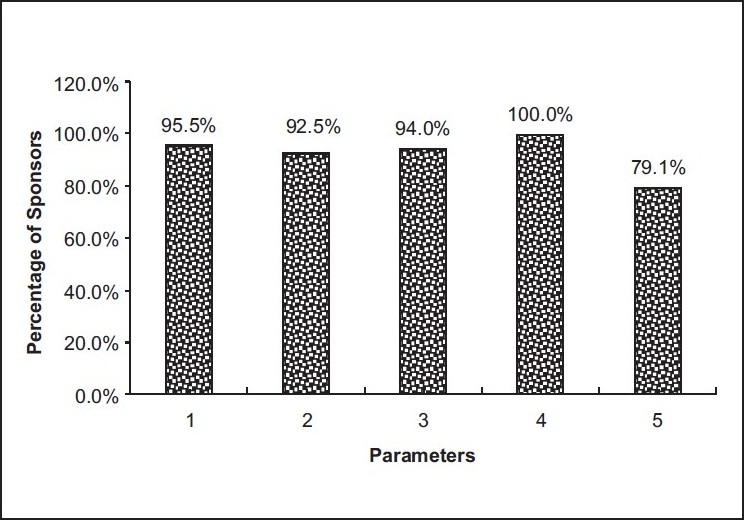
Parameters to conduct GCP clinical trials by investigator capabilities and experience

VII. Parameters for Investigator capabilities and experience to conduct GCP clinical trial according to the Sponsors’ opinion [Figure 4]:

1. 100.0% mentioned commitment/involvement of principal investigator (PI) in the study
2. 95.5% opined about clinical trial experience of the investigator
3. 94.0% stated availability of desired patient population
4. 92.5% referred to availability of GCP-trained study coordinator
5. 79.1% declared leader stature of the investigator in the desired therapeutic area

VIII. Parameters for facility available at trial site to conduct GCP clinical trial according to the Sponsors’ opinion:

1. 100.0% mentioned quality control (QC) /quality assurance (QA) procedure of the lab
2. 95.5% stated Ethics Committee’s Standard Operating Procedure (EC’s SOP) in compliance with International Conference on Harmonisation-Good Clinical Practice (ICH-GCP) and applicable regulatory guidelines and availability of dedicated space for drug storage, record-keeping and monitoring
3. 94.0% opined about post-study archival facility

IX. Parameters related to clinical trial conduct according to the Sponsors’ opinion:

1. 100.0% mentioned protocol adherence and GCP compliance
2. 92.5% stated meeting enrolment, and data management matrix
3. 79.1% declared cost-effectiveness

X. Importance of CROs in conducting clinical trials according to the Sponsors’ opinion:

1. 100.0% mentioned that CROs provide rapid, flexible access to additional research and development capacity/resources, product of outside sponsor’s core therapy areas and product at high risk
2. 98.4% stated risk management tool for high-risk, low-priority projects, CROs have better access to patients
3. 96.9% opined about sponsors transfer fixed costs to variable costs and access to enabling technology
4. 95.3% mentioned access to therapeutic expertise outside core areas

## DISCUSSION

According to the ECMs surveyed, the majority mentioned that scientific rationale of the study, protection of the rights and safety of the trial participants, clearance of the study project by DCGI, investigator’s qualification and clinical research experience are the most important parameters to conduct GCP clinical trials. They mentioned that their most important role in the clinical trial is to safeguard the right, safety, and wellbeing of all trial subjects and also to ensure a competent review of all the ethical aspects of the project proposals received as well as to execute the project free from any bias and influence that could affect its objectivity. ECMs stated that they follow all the principles while reviewing the clinical trial protocols. According to them the most important are principles of compliance, principles of privacy and confidentiality, and principles of voluntariness, informed consent and community agreement.

According to the investigators surveyed, they mentioned various parameters to conduct GCP clinical trial. They said that scientific rationale of the study and application of the study in the Indian context are the most important parameters of study design for conducting GCP clinical trial. Furthermore, they stated that scientific quality of protocol, CRF and ICD design are the most important parameters of project management (Sponsor/CRO). Finally, they opined that trial management capabilities, qualification and experience, communication skills and responsiveness are very important parameters of personnel quality (Clinical Research Associate or CRA/Monitor) for conducting GCP clinical trial.

The sponsor may be an individual, company, institution, or organization which takes responsibility for the initiation, management, and/or financing of a clinical trial. An individual who both initiates and conducts, alone or with others, a clinical trial, and under whose immediate direction the investigational product is administered to, dispensed to, or used by a subject. According to the sponsors surveyed, they mentioned that the commitment/involvement of the principal investigator (PI) in the study is most important for investigator’s capabilities and experience. In continuation of the detailed discussion, they stated that quality control/quality assurance procedure of the lab, protocol adherence and GCP compliance, rapid and flexible access to additional research and development capacity/resources, product of outside sponsor’s core therapy areas and product at high risk are the most important parameters for conducting GCP clinical trial.

## CONCLUSION

The study has identified a specific training need at the level of the individual stakeholder to perform a particular job function and the key objectives of the study are as follows: to identify the actual practical parameters in the Indian context important for the conduction of clinical trials (GCP) with respect to the different stakeholders, to determine the relative importance of these parameters as perceived by various stakeholders involved in clinical trials, and to identify the relative contributions of different stakeholders to the success/ satisfactory conduct of a clinical trial.

According to the stakeholders surveyed, the scientific rationale of the study, safeguarding the right, safety, and wellbeing of all trial subjects, principles of compliance, quality of protocol, CRF and ICD design, trial management capabilities, qualification and experience, and communication skills of personnel (CRA/Monitor), commitment/ involvement of principal investigator (PI) in the study, QC/QA procedure of the lab, protocol adherence and GCP compliance are the most important practical parameters which would contribute to the successful conduct of a GCP clinical trial.
